# Electroacupuncture Improves Clearance of Amyloid-*β* through the Glymphatic System in the SAMP8 Mouse Model of Alzheimer's Disease

**DOI:** 10.1155/2021/9960304

**Published:** 2021-08-26

**Authors:** Pei-zhe Liang, Li Li, Ya-nan Zhang, Yan Shen, Li-li Zhang, Jie Zhou, Zhi-jie Wang, Shu Wang, Sha Yang

**Affiliations:** ^1^First Teaching Hospital of Tianjin University of Traditional Chinese Medicine, Tianjin, China; ^2^National Clinical Research Center for Chinese Medicine Acupuncture and Moxibustion, Tianjin, China; ^3^Academy for Advanced Interdisciplinary Studies Peking University, Beijing, China; ^4^Tianjin Key Laboratory of Acupuncture and Moxibustion, Tianjin, China; ^5^Tianjin Academy of Traditional Chinese Medicine Affiliated Hospital, Tianjin, China; ^6^Key Laboratory of Cerebropathy Acupuncture Therapy of State Administration of Traditional Chinese Medicine, Tianjin, China

## Abstract

**Background:**

Memory loss and cognitive impairment characterize the neurodegenerative disorder, Alzheimer's disease (AD). Amyloid-*β* (A*β*) is the key factor that triggers the course of AD, and reducing the deposition of A*β* in the brain has been considered as a potential target for the treatment of AD. In clinical and animal studies, electroacupuncture (EA) has been shown to be an effective treatment for AD. In recent years, substantial evidence has accumulated suggesting the important role of the glymphatic system in A*β* clearance.

**Objective:**

The purpose of this study was to explore whether EA modifies the accumulation of A*β* through the glymphatic system and may thus be applied to alleviate cognitive impairments.

**Methods:**

Seven-month-old SAMP8 mice were randomized into a control group (Pc) and an electroacupuncture group (Pe). Age-matched SAMR1 mice were used as normal controls (Rc). Mice in the Pe group were stimulated on Baihui (GV20) and Yintang (GV29) for 10 min and then pricked at Shuigou (GV26) for ten times. EA treatment lasted for 8 weeks. In each week, EA would be applied once a day for the first five consecutive days and ceased at the remaining two days. After EA treatment, Morris water maze (MWM) test was used to evaluate the cognitive function; HE and Nissl staining was performed to observe the brain histomorphology; ELISA, contrast-enhanced MRI, and immunofluorescence were applied to explore the mechanisms underlying EA effects from A*β* accumulation, glymphatic system function, reactivity of astrocytes, and AQP4 polarization, respectively.

**Results:**

This EA regime could improve cognition and alleviate neuropathological damage to brain tissue. And EA treatment might reduce A*β* accumulation, enhance paravascular influx in the glymphatic system, inhibit the reactivity of astrocytes, and improve AQP4 polarity.

**Conclusion:**

EA treatment might reduce A*β* accumulation from the brain via improving clearance performance of the glymphatic system and thereby alleviating cognitive impairment.

## 1. Introduction

Alzheimer's disease (AD), a common degenerative disease of the central nervous system (CNS), is characterized by progressive memory impairment, aphasia, agnosia, and executive dysfunction, accompanied with a varying degree of changes in personality and behavior [1]. Amyloid *β* (A*β*) accumulation is a histopathological hallmark of AD [2–5]. In recent years, there is substantial evidence suggesting that the astrocyte-mediated brain-wide paravascular pathway may contribute to A*β* clearance [6, 7]. Such pathway is also referred as “glymphatic system” since the draining space resembles the peripheral lymphatic system. The glymphatic system enables cerebrospinal fluid (CSF) to enter the brain via periarterial spaces and move into the interstitium via aquaporin-4 (AQP4) located in the endfeet of perivascular astrocytes; it also drives the perivenous drainage of interstitial fluid (ISF) and its solutes, thereby clearing the brain of metabolic waste [8–12]. AQP4 is an important factor affecting the glymphatic system's functionality [6]. The increasing reactivity of astrocytes in the aging brain causes the loss of AQP4 polarization, and thus, A*β* clearance by the glymphatic system is reduced [7].

Electroacupuncture (EA), a combination of electrical stimulation and traditional acupuncture, is an important nondrug treatment for AD and is widely used in clinical practice [13]. EA has been shown to improve cognitive performance, neuropsychiatric symptoms, and the ability to perform daily activities in Alzheimer's patients [14, 15]. Previous experimental studies have shown that EA can improve cognitive deficits in animal models of AD [16, 17], and such benefit might contribute to reducing A*β* deposition and thereby delaying the pathological progression of AD from multiple pathways. For instance, EA intervention might upregulate the content of A*β*-degrading enzymes such as neprilysin (NEP) and insulin-degrading enzyme (IDE) in the brain, enhance the phagocytosis of A*β* by microglia, and improve the expression of A*β*-related transporters on the blood-brain barrier [18–21]. The glymphatic system is also an important clearance pathway of A*β*. However, no existing study demonstrates whether the glymphatic system might participant in the EA's effect on regulating A*β* deposition in AD.

**Figure 1:**
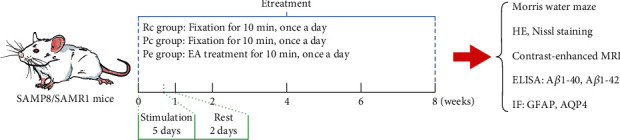
The process of this study.

Here, we investigated the effect of EA on the clearance of A*β* via the glymphatic system in AD model mice, to provide more scientific experimental support for the treatment of AD with electroacupuncture. In the light of a previous study [16, 20, 22, 23], senescence-accelerated mouse prone 8 (SAMP8) mice were used for the animal model of AD and the age-matched senescence accelerated mouse resistant 1 (SAMR1) mice as the control with normal aging phenotype. After EA treatment, the Morris water maze (MWM) test was applied to evaluate cognition, HE and Nissl staining to determine brain histomorphology, ELISA to ascertain the expression of A*β*, contrast-enhanced MRI for the assessment of glymphatic system function, and immunofluorescence to assess the reactivity of astrocytes and AQP4 polarization. The process of this study is described in Figure 1.

## 2. Material and Methods

### 2.1. Animals

7-month-old male SAMP8 and SAMR1 mice (30 ± 5 g) were provided by the SAM Breeding Center of the First Teaching Hospital of Tianjin University of Traditional Chinese Medicine (license number: SCXK (Jing) 2015-0003). All mice were housed under standard conditions at 23 ± 2°C and a 12 h light/dark cycle with free access to food and water. The Animal Ethics and Welfare Committee of Tianjin University of Traditional Chinese Medicine approved all procedures (TCM-LAEC2020028).

### 2.2. Animal Grouping and Electroacupuncture Treatment

Before the experiment began, all mice were initially screened through a water maze to remove those that were too sensitive or stupid. The remaining thirty SAMP8 mice were randomly divided into a SAMP8 control group (Pc) and SAMP8 electroacupuncture group (Pe). Fifteen SAMR1 mice were assigned to a SAMR1 normal control group (Rc).

**Figure 2:**
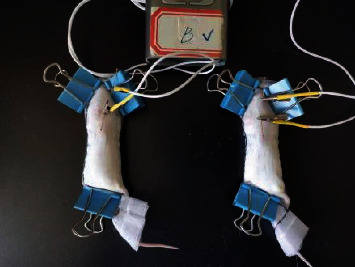
EA stimulation.

Based on our previous preliminary experiment, GV20, GV29, and GV26 were selected for EA regime. During EA stimulation, the mice were immobilized in self-made fixators (Figure 2). Location of these acupoints in mice was determined as follows [17]. GV20 is located halfway between the auricular apices; GV29 is located halfway between the medial ends of the two eyebrows; and GV26 is below the apex nasi, at one-third from the top of the midline of the cleft lip. Two sterile acupuncture needles were separately inserted downward horizontally at GV20 and upward horizontally at GV29, to a depth of 0.5 cm. Continuous-wave stimulation for 10 min at a frequency of 2 Hz (intensity 1 mA) was applied by a programmed apparatus (HANS-100A, Nanjing Jisheng Medical Technology, China) to which the needles were connected. Then, GV26 acupoint was pricked quick for 10 times with the needle at the end of EA treatment. The treatment lasted for a total of 8 weeks. In each week, EA would be applied once a day for the first five consecutive days and ceased at the remaining two days. The Pc group and Rc group did not receive EA stimulation, only the same fixation as the Pe group.

### 2.3. Morris Water Maze Test

Morris water maze (MWM) is a well-validated test for cognitive function. For the whole test period, the lab was maintained as a sound-insulated and low-light environment, while the objects in the room were kept at their original locations and other experimental conditions remained unchanged. After EA treatment, a circular water tank (80 cm in diameter and 50 cm in depth) divided into four equal quadrants (I, II, III, and IV) was used for MWM test; a circular escape platform (9 cm in diameter and 28 cm in height) was placed at the center of quadrant I. The tank was filled with warm water (23 ± 1°C) to a depth of 30 cm and edible melanin to dye the water black. Visual cues of various shapes were located on the side of each quadrant of the tank, in plain sight of the mice. A digital camera connected with an image acquisition system was used to automatically track all trials. The MWM test was performed as two stages. (1) To evaluate changes in learning ability, each animal was subjected to a trial with a hidden platform. In order to familiarize mice with the test environment, the mice used in the experiment were separately allowed to swim freely in the water tank (without platform) for 60 seconds on the day before being subjected to the hidden platform trial. Throughout the trail, mice in each group were placed in the water at a fixed position in quadrant III while the submerged platform remained fixed in location in quadrant I. If the tested animal found the hidden platform within 60 seconds and stayed on it for at least 5 seconds, we recorded the escape latency time (expressed as the time spent swimming to find the platform). If the animal failed to find the platform within 60 seconds, it was placed on the platform for 10 seconds, allowing it to observe and memorize the location, and the escape latency time was recorded as 60 seconds. The trial with a hidden platform was repeated daily for five consecutive days. (2) We conducted a probe trial on day six to measure the ability of the mice to maintain long-term memories with the hidden platform removed. The frequency with which the platform's former location was crossed was recorded together with the time spent in the target quadrant (I) during the trial.

### 2.4. HE Staining

The mice were perfused with 0.01 M phosphate-buffered saline (PBS) and 4% paraformaldehyde successively under isoflurane gas anesthesia (isoflurane, RWD Life Science Co., Ltd., China), and then, the brains were dissected and postfixed for 24 hours in 4% paraformaldehyde. To observe pathologic morphological changes with HE staining, standard paraffin tissue sections of the brain tissue were prepared (LEiCA RM2245, China). The section thickness was 5 *μ*m.

### 2.5. Nissl Staining

Brains were immersed in 4% paraformaldehyde, embedded in paraffin, and then cut into coronal sections for Nissl staining. Briefly, the sections were deparaffinized in xylene, gradually rehydrated using alcohol, and treated with Nissl staining solution (Bioss, Beijing, China) for 5 min. Then, sections were mounted in neutral balsam and examined by light microscopy (Olympus, DP26). The number of Nissl bodies observed in three fields of the hippocampus and cortex was recorded. The IPP6.0 software was used for analyzation and quantification.

### 2.6. ELISA

The mice were euthanized by cervical dislocation under isoflurane gas anesthesia. The brains were removed immediately, followed by isolation of the hippocampus and cortex. Using mouse A*β*1-40 and A*β*1-42 ELISA kits (Meimian, Jiangshu, China), the A*β* levels were measured following the manufacturer's instructions.

### 2.7. Contrast-Enhanced MRI

To measure the dynamic cerebrospinal-interstitial fluid (CSF-ISF) exchange, 3D T1-weighted MR images (T1WIs) with Gd-DTPA contrast agent were used following a protocol for contrast-enhanced MRI (CE-MRI) [9]. The mice were anesthetized and fixed in a stereotaxic apparatus, then dissected to expose the cisterna magna. A total of 7 *μ*l Gd-DTPA was delivered to the cisterna magna at an infusion rate of 2 *μ*l per minute for each mouse. MRI (Bruker, Germany) measurements were performed 10, 20, 30, 40, and 50 min after injection; anesthesia was maintained using isoflurane gas. The signal intensity of regions of interest (ROIs), including the pituitary recess, olfactory arterial complex, and olfactory bulb, was recorded.

### 2.8. Immunofluorescence

Paraffin sections were treated with 1% BSA for 30 min. Subsequently, sections were incubated overnight in the dark using rabbit anti-GFAP (Bioss, Beijing, China) and mouse anti-AQP4 (Bioss, Beijing, China) primary antibodies at the temperature of 4°C. They were then incubated with the corresponding secondary antibodies. The nuclei were stained using DAPI (Bioss, Beijing, China). The slides were examined using a fluorescence microscope (Olympus, BX51).

### 2.9. Quantitative Analysis of AQP4 Water Channel Polarization

Polarization of the astrocytic AQP4 was analyzed using immunofluorescence images, with reference to a previous study [24]. Histological sections labeled for GFAP and AQP4 were subjected to color channel separation. The overall area of AQP4 immunoreactivity was defined by the low-stringency threshold, whereas the high-stringency threshold demarcated the area of intense AQP4 immunoreactivity found in the endfeet of the astrocytes. “AQP4 polarity” was defined as the ratio of low-stringency area to high-stringency area. Higher AQP4 polarity means that a greater proportion of immunoreactivity is restricted to perivascular regions, whereas a lower proportion shows that the distributed immunoreactivity is restricted to the astrocytes' soma.

### 2.10. Statistical Methods

All results are reported using their mean and SD. Between-group differences were assessed by one-way analyses of variance (ANOVA), followed by tests of least-significant difference (LSD; equal variances assumed) or Dunnett's T3 (equal variances not assumed). For all comparisons, a significance level of *P* < 0.05 was assumed. All analyses were done with SPSS version 23.0.

## 3. Results

### 3.1. EA Improves Learning Ability and Memory in SAMP8 Mice

**Figure 3:**
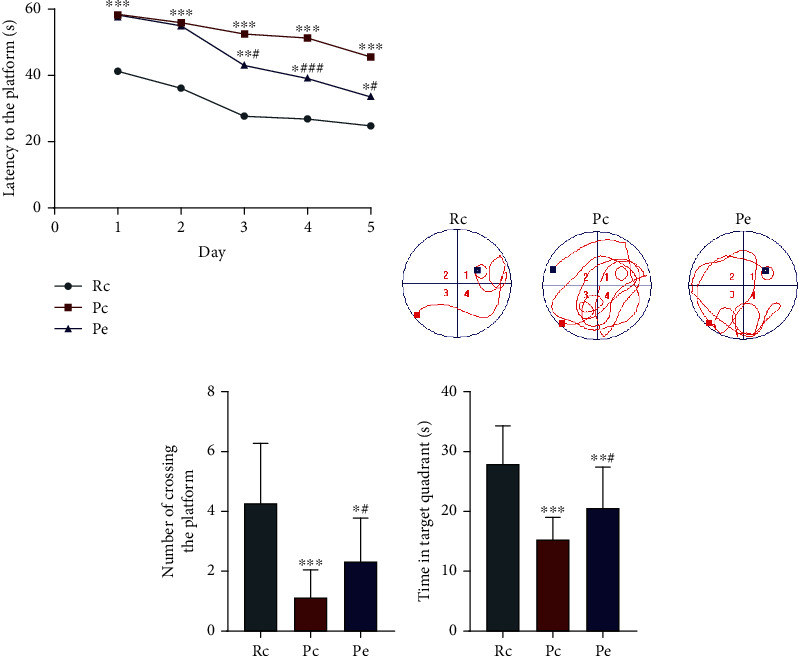
EA improved the ability of learning and memory of SAMP8 mice: (a) the escape latency time; (b) representative swim paths of each group in the hidden platform trial; (c) number of crossing the platform; (d) time in target quadrant. Data are expressed as the mean ± SD (*n* = 15 in each group). ^∗^*P* < 0.05 and ^∗∗∗^*P* < 0.001 versus the Rc group; ^#^*P* < 0.05 and ^###^*P* < 0.001 versus the Pc group.

The escape latency time in the hidden platform trial displayed a downward trend in each group as the number of training days increased. Compared with the Rc group, the Pc group revealed a longer escape latency time (*P* < 0.001), and the seven-month SAMP8 mice exhibited an obvious learning disability. On days 1 and 2 of the hidden platform trial, the Pc group and the Pe group did not differ statistically. After days 3, 4, and 5, the escape latency time in the Pe group decreased significantly compared to that in the Pc groups (*P* < 0.001 or *P* < 0.05), demonstrating that EA improved the learning ability of SAMP8 mice (Figure 3(a)). Typical swimming traces of each group reflected the search strategies in the hidden platform trial (Figure 3(b)). After training the trial for five days, the probe trial was utilized to test whether the memory was maintained. As more time was spent in the quadrant where the platform used to be and the more times the platform was crossed, the higher the level of memory retention was assumed to be. In this trial, the time spent in the target quadrant and the number of times that the platform was crossed were found to be significantly less in the Pc group than in the Rc group (*P* < 0.001), and the number is greater in the Pe group than in the Pc group (*P* < 0.05). These data suggest that memory was obviously disabled in the seven-month SAMP8 mice and that EA improved memory preservation capability (Figures 3(c) and 3(d)).

### 3.2. EA Alleviates Neuropathological Injury of Brain Tissue in SAMP8 Mice

**Figure 4:**
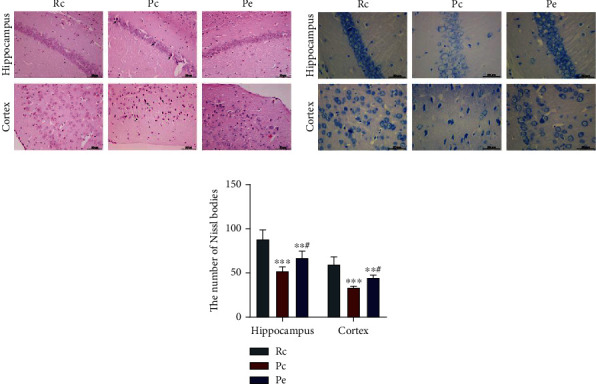
EA alleviated neuropathological injury of brain tissue in SAMP8 mice: (a) HE staining, scale bars = 50 *μ*m; (b) Nissl staining, scale bars = 20 *μ*m; (c) the number of Nissl bodies in the hippocampus and cortex. Data are expressed as the mean ± SD (*n* = 4 in each group). ^∗∗^*P* < 0.01 and ^∗∗∗^*P* < 0.001 versus the Rc group; ^#^*P* < 0.05 versus the Pc group.

Among all pathological changes of AD, neuron injury should be the basic one [25]. As shown by HE staining, no abnormalities were observed on in the hippocampus and cortex of SAMR1 mice in the Rc group since the structures of neurons were complete, dense, and orderly. Obvious pathological changes were observed in brain tissue of the Pc group in morphological and structural: neuron arrangement was loose, the nuclei had shrunk and were deeply stained, and fibrous tangles were visible (black arrow; Figure 4(a)). To a certain degree, pathological changes in neurons at the hippocampus and cortex area of the Pe groups were alleviated compared with those of the Pc group, where the neuron arrangement was orderly, with a small number of abnormalities in the morphology and structure of neurons. Nissl bodies are a characteristic structure of neurons, and its number is a gold index to reflect the functional state of neurons [26]. Compared with the Rc control group, Nissl bodies in the Pc group were arranged sparsely, and there were significantly fewer Nissl bodies in the hippocampus and cortex (*P* < 0.001). After EA treatment, the number of Nissl bodies was higher in the Pe group than in the Pc group (*P* < 0.05; Figures 4(b) and 4(c)). These results indicate that neuropathological injury had been observed in the brains of seven-month SAMP8 mice and that EA could alleviate this injury in the brain tissue of SAMP8 mice.

### 3.3. EA Reduces A*β* Accumulation in SAMP8 Mice

**Figure 5:**
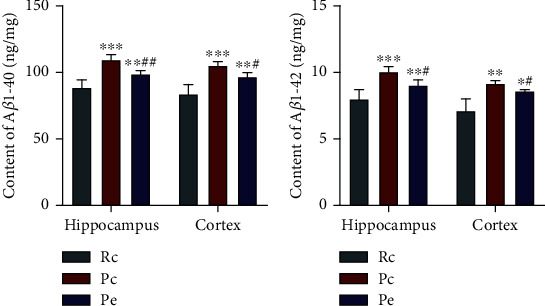
EA reduced A*β* accumulation in SAMP8 mice: (a) content of A*β*1-40 in the hippocampus and cortex; (b) content of A*β*1-42 in the hippocampus and cortex. Data are expressed as the mean ± SD (*n* = 6 in each group). ^∗∗^*P* < 0.01 and ^∗∗∗^*P* < 0.001 versus the Rc group; ^#^*P* < 0.05 and ^##^*P* < 0.01 versus the Pc group.

ELISA was applied to investigate the effects of EA on A*β* production (A*β*1-40 and A*β*1-42) in the brain of SAMP8 mice. The results showed that the contents of A*β*1-40 and A*β*1-42 in both the hippocampus and cortex of the Pc group were significantly higher than those of the Rc group (*P* < 0.001 and *P* < 0.01, respectively). Compared with the Pc group, the contents of both A*β*1-40 and A*β*1-42 were reduced significantly in the Pe group (*P* < 0.01 and *P* < 0.05, respectively; Figures 5(a) and 5(b)). These data suggest that accumulation of A*β* in the brain was increased in SAMP8 mice at seven months, and EA might reduce A*β* accumulation in the brain.

### 3.4. EA Improves Clearance Function of the Glymphatic System in SAMP8 Mice

**Figure 6:**
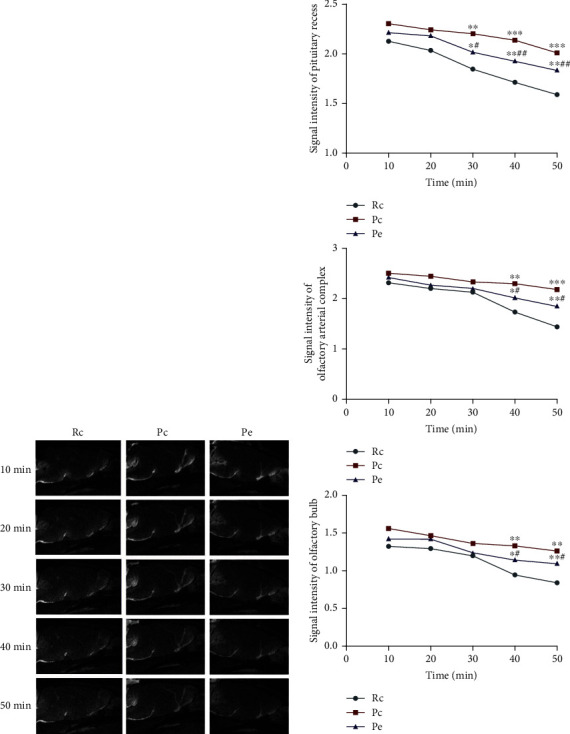
EA improved glymphatic system function in SAMP8 mice: (a) time-dependent anatomical routes of paravascular influx in the glymphatic system; (b) signal intensity of ROIs. Data are expressed as the mean ± SD (*n* = 3 in each group). ^∗^*P* < 0.05, ^∗∗^*P* < 0.01, and ^∗∗∗^*P* < 0.001 versus the Rc group; ^#^*P* < 0.05 and ^##^*P* < 0.01 versus the Pc group.

Contrast-enhanced MRI allows the assessment of the brain-wide CSF-ISF exchange in glymphatic system. After contrast agent was injected into the subarachnoid space of the cisterna magna, it would pass through specific paravascular pathways before entering the brain parenchyma along with CSF, where contrast agent and CSF were intermingled with fluid in the interstitial compartments. Dynamic time series of T1-weighted MRIs could clearly reveal the different anatomical paths followed over time by the paravascular influx in the glymphatic system; thus, paramagnetic contrast agent injected into the cisterna magna was transported along the basilar artery and appears in the pituitary recess, from where it was conveyed along the olfactory arterial complex and into the olfactory bulb (Figure 6(a)). Compared with the Rc group, the signal intensity of all ROIs in the Pc group showed a higher trend, indicating that the retention concentration of Gd-DTPA was higher. Starting from the time of Gd-DTPA injection, the signal intensity in the pituitary recess was significantly higher in the Pc group than in the Rc group after 30 min, while that in the Pe group was significantly lower than in the Pc group. The signal intensity at the olfactory arterial complex and olfactory bulb was significantly higher in the Pc group than in the Rc group for 40 min after Gd-DTPA injection, while in the Pe group, it was significantly lower than in the Pc group (*P* < 0.05; Figure 6(b)). The overall effects in the Pc group versus the Rc group reflects that reduced clearance of Gd-DTPA during CSF–ISF exchange caused the higher residual concentrations, the suppressed glymphatic system, and the reduction in its ability to clear and that EA could accelerate the CSF-ISF exchange, improve the function of the glymphatic system in SAMP8 mice, and promote the clearance of material from the brain's parenchyma.

### 3.5. EA Inhibits the Reactivity of Astrocytes and Improves AQP4 Polarity

**Figure 7:**
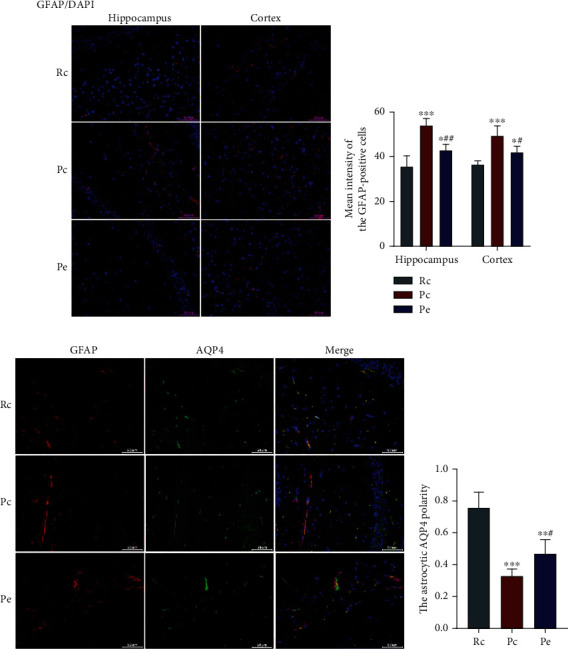
EA inhibited the reactivity of astrocytes and improved AQP4 polarity. (a) GFAP-positive cells were spread in each group, scale bars = 50 *μ*m. (b) The mean fluorescence intensity of GFAP-positive cells in the hippocampus and cortex. (c) Immunofluorescence: double-labeling of GFAP and AQP4, scale bars = 50 *μ*m. (d) Astrocytic AQP4 polarity. Data are expressed as the mean ± SD (*n* = 4 in each group). ^∗^*P* < 0.05, ^∗∗^*P* < 0.01, and ^∗∗∗^*P* < 0.001 versus the Rc group; ^#^*P* < 0.05 and ^##^*P* < 0.01 versus the Pc group.

In the brain, impairment of the glymphatic system and the depolarization of AQP4 in reactive astrocytes are closely associated [7]. Astrocyte activation in the brain's parenchyma and AQP4 polarization were evaluated to clarify how EA treatment restores the glymphatic system's function. As shown by immunofluorescence, GFAP-positive astrocytes were present in the cortex and hippocampus of SAMR1 and SAMP8 mice. In the Pc group, the mean fluorescence intensity of GFAP-positive cells in the hippocampus and cortex was significantly higher than that in the Rc group (*P* < 0.001), and the mean fluorescence intensity of GFAP-positive cells in the hippocampus and cortex was significantly lower in the Pe group than in the Pc group (*P* < 0.01 and *P* < 0.05, respectively; Figures 7(a) and 7(b)).

AQP4, the main component of water channel proteins expressed by astrocytes, is polarized in the perivascular astrocytic endfeet of the healthy brain, but not of the aging brain. The displacement of AQP4 from the endfeet of astrocytes to their soma is, in part, associated with failure of the glymphatic system-paravascular pathway [7]. We therefore explored the polarization of AQP4 in the brain of SAMR1 and SAMP8 mice. In the Rc group, AQP4 was expressed well all over the perivascular region, where it sheathed the astrocytic endfeet, whereas in the Pc group, it was located wrongly in the soma of astrocytes. In the Pe group, AQP4 was less widespread in the astrocytic soma, being mostly distributed around endfeet. The value of AQP4 polarity was analyzed following a previous study [24] and was assessed in SAMR1 and SAMP8 mice by the ratio between low-stringency area (total area of AQP4 immunoreactivity) and high-stringency area (area of intense AQP4 immunoreactivty around the perivascular endfeet) in an image. As a result, astrocytic AQP4 polarity was shown to be significantly lower in the Pc group than in the Rc group (*P* < 0.001) and higher in the Pe group than in the Pc group (*P* < 0.05; Figures 7(c) and 7(d)). The above result suggests that EA inhibited the reactivity of astrocytes and improved AQP4 polarity, triggering an improvement of the glymphatic system's function in the SAMP8 brain.

## 4. Discussion

At present, the treatment of AD is mainly based on antipsychotic drug therapy, but more and more studies have shown that antipsychotic drug therapy can increase the risk of death in Alzheimer's patients [27]. Therefore, nonpharmaceutical intervention for Alzheimer's disease has attracted more and more attention, including cognitive behavioral therapy (CBT), music therapy, transcranial magnetic stimulation (TMS), and acupuncture therapy. CBT and music therapy can reduce psychological and emotional problems such as depression and anxiety, but there are few reports on whether these two therapies can improve the cognition of AD patients [28, 29]. TMS mainly improves cognitive function by changing the excitability of local cerebral cortices [30]. However, TMS devices may cause side effects such as headache and even epilepsy during treatment due to the electrical characteristics of high voltage, high current, and strong magnetic field [31, 32]. Acupuncture, one of the most important nonpharmaceutical treatments that originated from traditional Chinese medicine, has a long history in the treatment of dementia in China. EA, a combination of traditional acupuncture and electrical stimulation [13], is safe and highly accessible. Clinical studies have shown that EA is one of the effective techniques for treating AD [33]. EA can effectively improve the overall intelligence and cognitive function of patients and alleviate various emotional symptoms of AD for a long time, with few negative side effects [14, 15]. In conclusion, EA has a wide range of action and is relatively safe among various nondrug treatments, so EA was selected as an intervention method in this study. In previous studies, mechanisms of EA for cognitive function have been explored from different aspects in animal models of AD. The experiments have demonstrated that EA can effectively improve memory function and learning by inhibiting A*β* deposition, tau phosphorylation, and neuron loss [20, 21, 34–36].. Besides, a previous study also has shown that EA can promote A*β* clearance through A*β*-degrading enzymes, microglia phagocytosis, and blood-brain barrier transport, thereby delaying cognitive impairment in animal models of AD [18, 19, 37, 38]. The glymphatic system is closely related to the clearance of A*β*, but the research on the mechanism of EA regulating A*β* targeting the glymphatic system is still insufficient. Therefore, our study was designed to fill that gap for better understanding the mechanism of EA in treating AD.

According to the theory of traditional Chinese medicine, the trunk and branches of Governor Vessel (GV) are all closely correlated with the brain and if lesions occur in the brain, the GV is recommended as a preferred treatment option, and therefore, the acupoints in GV are frequently used in the treatment of CNS diseases. In our study, GV20, GV29, and GV26 were selected for the treatment since these three acupoints of GV could supplementarily combine to regulate the spirit, activate local collaterals, calm the frightened and awaking mind, awake the brain, and open orifices, respectively, therefore enhancing cognitive function and benefiting intelligence. The efficacy of such combination has been confirmed experimentally by many previous studies [17, 35, 39, 40]. Progressive cognitive impairment is the most common symptom of AD patients [41], and the progressively low cognitive ability of AD patients is closely linked to neuron loss in the brain [42]. Consistent with previous studies, our results of MWM test and HE and Nissl staining all demonstrated that EA could improve the cognitive ability to learn, memorize, and explore the target platform and has a protective effect on neurons. Our data supported that such EA regime is effective in the treatment of AD animal models.

The hypothesis of “amyloid cascade for Alzheimer's disease” proposes that excessive accumulation of A*β* is the initiator and core link of the AD progression. In AD patients, the excessive accumulation of A*β* in the brain parenchyma forms senile plaque (SP), which can stimulate hyperphosphorylated tau aggregation, form NFTs, increase free radical release, and enhance oxidative stress response, which leads to synaptic degeneration and neuron death and ultimately triggers AD [43]. A variety of previous evidences have shown that EA can improve cognitive impairment in animal models of AD by reducing A*β* accumulation [20, 21]. Consistent with previous studies, our results of ELISA demonstrated that A*β* accumulation in the brain decreased significantly after EA treatment. Furthermore, how EA decreases A*β* accumulation of AD model was also explored.

In vivo, A*β* is mainly cleared from the brain by related degrading enzymes, glial cell phagocytosis, and blood-brain barrier transport [44]. In recent years, it is suggested that metabolites closely related to AD such as A*β*, Apolipoprotein E (ApoE), and tau protein [45, 46] can be cleared through the paravascular pathway in addition to the classical way of clearance [6]. This is the emerging concept of “glymphatic system.” Dysfunction of the glymphatic system has been linked experimentally to A*β* accumulation [47]. Previous studies have shown that EA could reduce A*β* accumulation in the brain by upregulating the expression of A*β*-degrading enzymes such as neprilysin (NEP) and insulin-degraded enzyme (IDE) [18, 37], enhancing the A*β* uptake by microglia [38], and improving the expression of A*β*-related transporters such as low-density lipoprotein receptor-related protein-1 (LRP1) under the blood–brain barrier (BBB) [19]. However, evidence on the clearance of glymphatic system is lacking.

Iliff et al. found that glymphatic pathway function could be measured using dynamic contrast-enhanced MRI following intrathecal contrast agent administration. Dynamic contrast-enhanced MRI provides an intuitive approach to characterize both the kinetics and spatial distribution of paravascular CSF-ISF exchange throughout the whole brain [6]. The method has been widely used in research on the mechanism of the glymphatic system [48–51]. We used contrast-enhanced MRI to show the paravascular pathway and found that EA accelerated paravascular CSF-ISF exchange and significantly improved the function of glymphatic system. Reactive astrogliosis is a conspicuous feature of aging and the injured brain [7, 46, 52], and reactive astrocytes lead directly to withdrawal of AQP4 from the endfeet to the soma of astroglia, thus affecting the clearance function of the glymphatic system [7]. Here, EA was found to inhibit the reactivity of astrocytes and maintain the preferential location of AQP4 in the endfeet. Similar to our results, previous studies have demonstrated that the paravascular pathway's function is improved, and A*β* accumulation and impaired spatial memory cognition reduced by overexpression of Slit2 in the aging brain [53]. Based on these data, we propose that EA inhibits the reactivity of astrocytes and maintains AQP4 polarity, which improves the glymphatic system's function, thereby promoting A*β* clearance and consequently improving learning and the memory ability in SAMP8 mice.

There are some limitations in this study. In our study, dynamic contrast-enhanced MRI was used to demonstrate the EA could improve the overall clearance function of the glymphatic pathway, and it is suggested that when the overall clearance efficiency is improved, the clearance rate of A*β* could also be improved. However, direct evaluation of A*β* clearance from paravascular pathways is lacking. Previous studies have found that the neuroinflammation involved by activated astrocytes plays an important role in the pathogenesis of AD [54]. The results of this study showed that the injury of the glymphatic system in the animal model of AD is closely related to the increasing reactivity of astrocytes. Therefore, in the later stage, in-depth studies can be carried out based on the relationship between the function of the glymphatic system and neuroinflammation, as well as the reasons for the increasing reactivity of astrocytes and the effects on the brain tissue. Meanwhile, this study was conducted in order to further explore the regulation mechanism of EA on the glymphatic system and inflammatory response.

## 5. Conclusion

In summary, EA treatment can improve cognitive impairment in AD animal models, and its mechanism may involve the reduction of A*β* accumulation and improvement of the glymphatic system. This may provide new direction for further exploration of the mechanism of EA for the treatment of AD.

## Data Availability

The data used to support the findings of this study are available from the corresponding author upon request.
